# Synthesis and Biological Evaluation of Ginsenoside Compound K Derivatives as a Novel Class of LXRα Activator

**DOI:** 10.3390/molecules22071232

**Published:** 2017-07-24

**Authors:** Yan Huang, Hongmei Liu, Yingxian Zhang, Jin Li, Chenping Wang, Li Zhou, Yi Jia, Xiaohui Li

**Affiliations:** 1 Institute of Materia Medica and Department of Pharmaceutics, College of Pharmacy, Third Military Medical University, Shapingba, Chongqing 400038, China; huangyanoo8@126.com (Y.H.); hongmeiliu0819@126.com (H.L.);15095839731@163.com (Y.Z.); ioulj@tmmu.edu.cn (J.L.); wangchenping.0219@aliyun.com (C.W.); 2 Department of Pharmacy, Xinqiao Hospital & The Second Affiliated Hospital, Third Military Medical University, Shapingba, Chongqing 400037, China; zhouli1007@126.com

**Keywords:** atherosclerosis, LXRα, ginsenoside compound K, derivatives, reverse cholesterol transport

## Abstract

Compound K is one of the active metabolites of *Panaxnotoginseng* saponins, which could attenuate the formation of atherosclerosis in mice modelsvia activating LXRα. We synthesized and evaluated a series of ginsenoside compound K derivatives modified with short chain fatty acids. All of the structures of this class of ginsenoside compound K derivative exhibited comparable or better biological activity than ginsenoside compound K. Especially structure **1** exhibited the best potency (cholesteryl ester content: 41.51%; expression of ABCA1 mRNA: 319%) and low cytotoxicity.

## 1. Introduction

Atherosclerosis is a fundamental pathological process for some severe cardiovascular diseases, including stroke, coronary artery disease, and peripheral vascular disease, and contributes to one-fifth of all deaths in the world [1]. Generally practiced pharmacologic therapies for atherosclerosis, such as statins and fibrates, are targeted for down-regulation of cholesterol and/or triglyceride levels. Despite these lipid-lowering treatments being used for decades, serious cardiovascular diseases induced by atherosclerosis are still the leading cause of death in the developed world [2]. Therefore, further development of effective therapeutic approaches is desirable.

Our previous results have showed that *Panaxnotoginseng* saponins exhibit significant athero-protective effects, and the mechanism was associated with LXRα activation [3,4]. LXRα is a nuclear receptor protein and plays an important role in the regulation of cholesterol homeostasis and inflammation. LXRαregulates the reverse cholesterol transport process through the expression of down-stream proteins, such as ABCA1 and ABCG1. Thus, LXRα is considered a potential target for atherosclerosis therapy [5]. Ginsenoside compound K is one of the active metabolites of *Panaxnotoginseng* saponins [6]. Studies have indicated that ginsenoside compound K has multiple pharmacological activities, including inhibition of the proliferation of cancer [7,8] and smooth muscle cells [9], activation of glucocorticoid receptors [10], anti-inflammation [11], and so on. Besides the above biological effects, our previous study has shown that ginsenoside compound K could attenuate the formation of atherosclerosis in mice via activating LXRα [12], without presentation of the serious side effects caused by synthetic unspecific LXRs agonists, such as the elevation of plasma triglycerides [13] and liver steatosis [14]. The results indicated that ginsenoside compound K might have the potential to be a new effective structure for atherosclerosis therapy. However, there are still some issues with compound K pharmacological profiles, and poor water solubility is one of the major challenges [15].

Therefore, we designed and synthesized a series of ginsenoside compound K derivatives by introducing short chain fatty acid into the carbohydrate chain at C-3, C-18, C-32, C-33, C-34 and C-36. Since the formation of foam cells plays a key role in atherosclerosis, we detected the biological activities of the derivatives in foam cell model.

## 2. Results and Discussion

### 2.1. Water Solubility Measurements

**Figure 1:**
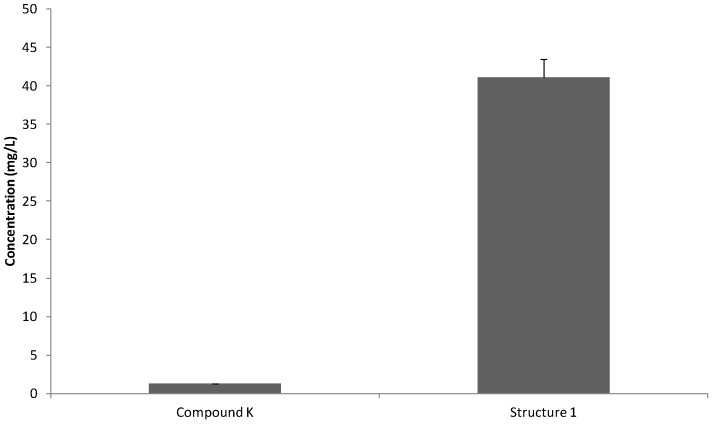
Water solubility measurements of structure **1** and compound K. Compound K and structure **1** were dissolved in ddH_2_O and determined by HPLC. Data were presented as mean ± SEM (*n* = 3).

The concentration of ginsenoside compound K and structure **1** in ddH_2_O was determined by HPLC. The results show that the mean water solubility of structure **1** (41.14 mg/L) was significantly higher than ginsenoside compound K (1.23 mg/L) (Figure 1).

### 2.2. Inhibition of the Formation of Foam Cells

**Figure 2:**
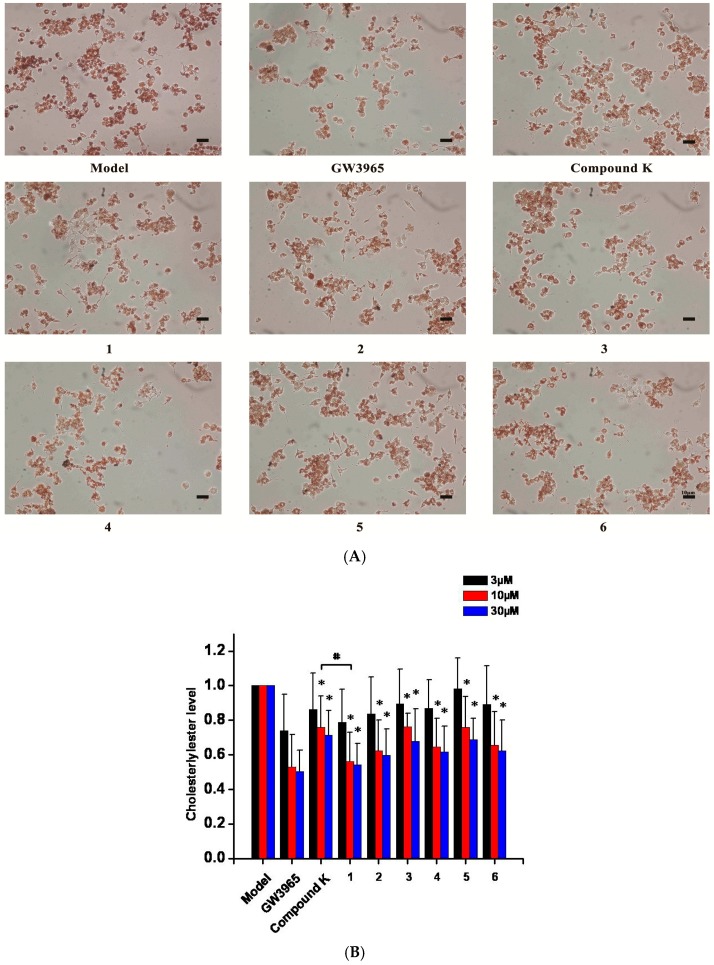
Effects of ginsenoside compound K derivatives on the formation of macrophage-derived foam cells. Foam cells were administrated by GW3965 (10 µM) and ginsenoside compound K derivatives (10 µM) were stained by Oil Red O. The visible red-stained lipid drops were captured under microscope (**A**). Oil Red O stained cellular cholesteryl ester was extracted from administrated foam cells and detected by the optical density values (**B**). Data were presented as mean ± SEM (*n* = 8) and analyzed by ANOVA (analysis of variance) with Dunnett’s post-hoc analysis. * *p* < 0.05 vs. Model. # *p* < 0.05 vs. Compound K.

Compared with the model group (treated with a bland DMSO solution), treatments with ginsenoside compound K and ginsenoside compound K derivatives (10, 30 µM) caused a significant decrease in lipid deposition which was red stained by Oil Red O in macrophage-derived foam cells (Figure 2A), consistent with the results of the quantity measurements of cellular cholesteryl ester (Figure 2B). The cholesteryl ester contents of the foam cells treated with structure **1** (10 µM) were significantly down-regulated compared with the cells treated with ginsenoside compound K. These results indicated that structure **1** presented better biological effects than ginsenoside compound K.

### 2.3. Effects on ABCA1 mRNA Expression

**Figure 3:**
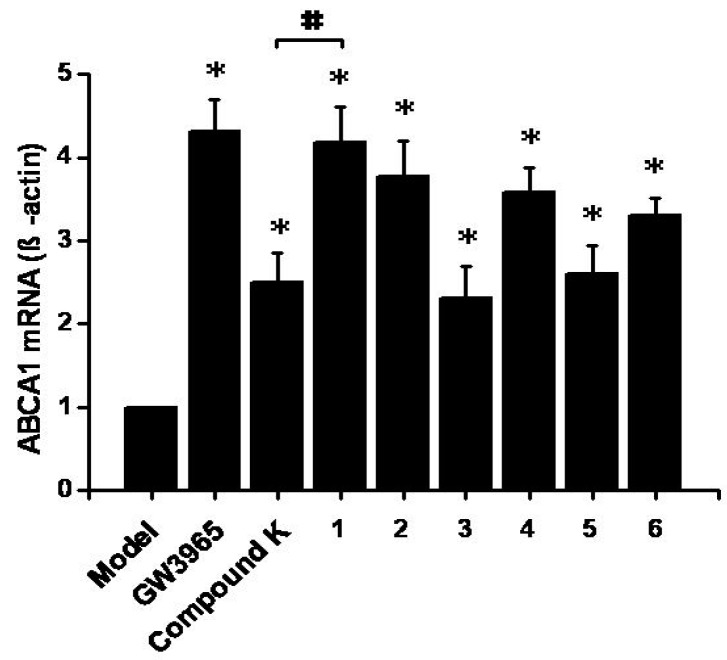
Effects of ginsenoside compound K derivatives on ABCA1 mRNA expression. RAW264.7 cells were treated with ginsenoside compound K derivatives in 10 μM. Expression level of ABCA1 mRNA was detected by the real-time PCR (polymerase chain reaction) method, and results were calculated relative to the control group. Data were presented as mean ± SEM (*n* = 5) and analyzed by ANOVA with Dunnett’s post-hoc analysis. * *p* < 0.05 vs. Control. # *p* <0.05 vs. Compound K.

Increasing the mRNA expression of ABCA1, which plays a critical role in reverse cholesterol transport, can cause a reduction in the formation of foam cells. Compared with the control group, ginsenoside compound K, structures **1**, **2** and **4** could increase the expression of ABCA1 mRNA by 151%, 319%, 278% and 259%, respectively (Figure 3). There was statistical difference in ABCA1 mRNA expression between the structure **1** group and the compound K group.

### 2.4. Luciferase Reporter Assay

**Figure 4:**
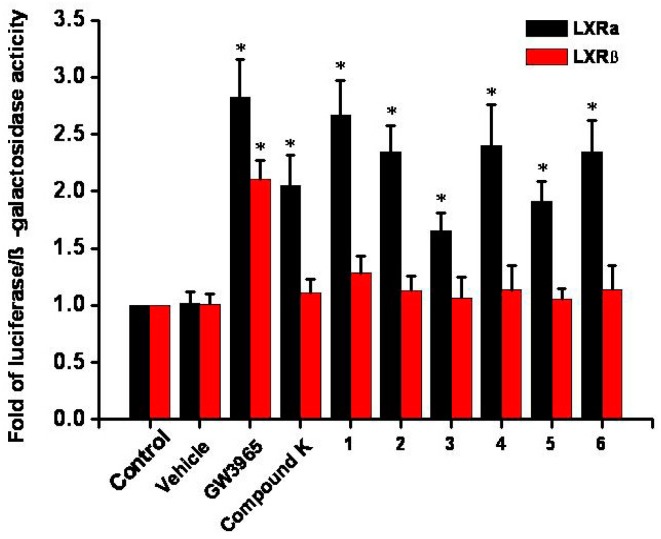
Effects of ginsenoside compound K derivatives on LXRα and LXRβactivation in HEK293. The results were showed as relative luciferase activity (fold difference compared to the control group). Ginsenoside compound K derivatives (10 µM) showed an elevation of luciferase activity for LXRα at different levels. Among them, structure **1** showed significant activation of LXRα. These structures did not show significant activation of luciferase activity for LXRβ. Data are presented as mean ± SEM (*n* = 3, each in duplicate), and analyzed by ANOVA with Dunnett’s *post-hoc* analysis. * *p* < 0.05 vs. Control.

The effects of ginsenoside compound K derivatives on LXRα and LXRβ activation in HEK293 cell line were detected. Ginsenoside compound K presented significant activation of LXRα (2.05 fold), consistent with our previous results. Ginsenoside compound K derivative structures **1**–**6**, showed elevation of luciferase activity for LXRα at different levels (Figure 4). Among them, structure **1** showed the highest activation of LXRα (2.67 fold), consistent with the results of the effects on the formation of foam cells and ABCA1 mRNA experiments. Ginsenoside compound K and the derivatives did not show significant activation of LXRβ.

### 2.5. Cell Toxicity

**Figure 5:**
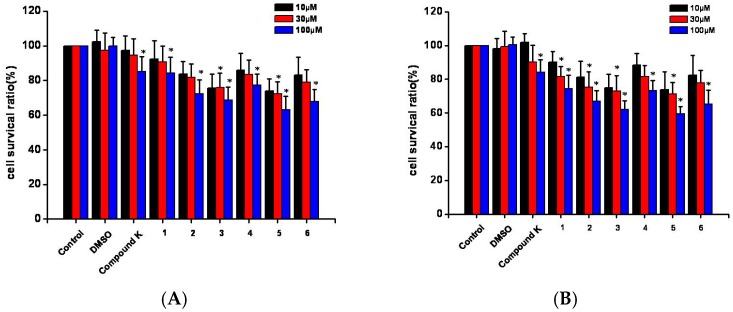
Cell toxicity of ginsenoside compound K derivatives. RAW264.7 (**A**) and HUVEC (**B**) cells were treated with ginsenoside compound K derivatives in different concentrations. Cell survival ratio was measured by the CCK-8 method, and results were calculated relative to the control group. Data were presented as mean ± SEM (*n* = 3) and analyzed by ANOVA with Dunnett’s *post-hoc* analysis. * *p* < 0.05 vs. Control.

Compared with the control group, ginsenoside compound K derivative structures **3** and **5** significantly decreased the survival ratios of RAW264.7 cells in 10 and 30 μM (Figure 5A). Compared with control group, structure **1** (30 μM), structure **2** (10 and 30 μM), structure **3** (10 and 30 μM) and structure **5** (10 and 30 μM) significantly decreased the survival ratios of HUVEC (human umbilical vein endothelial cell) (Figure 5B). All the structures and ginsenoside compound K showed significant toxic effects in 100 μM. The results of structure **3** and **5** might present more toxic effects.

### 2.6. Compound K and Structure ***1*** Dock into the LXRα 

**Figure 6:**
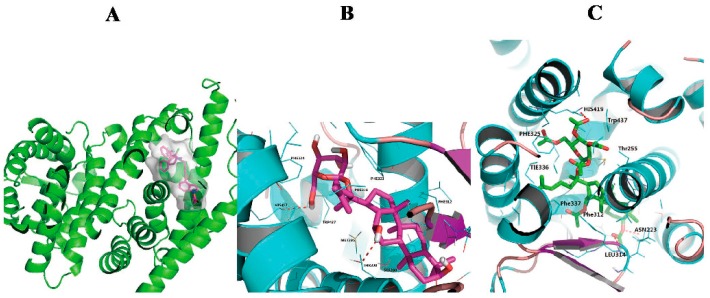
(**A**) The ginsenoside compound K ligand occupies a proportionally large volume of the cavity space within the ligand-binding pocket of LXRα; (**B**) A hydrogen bond was predicted between the hydroxyl group at C-36 of compound K and the carboxyl group of His-417, and the hydroxyl group at C-12 of ginsenoside compound K formed a hydrogen bond with the hydroxyl group ofThr-300; (**C**) the ester group at C-3 of ginsenoside compound K derivatives structure **1** formed a hydrogen bond with the amide group of Asn223, and the carboxyl group at C-3 of **1** formed a hydrogen bond with the amide group ofLeu314.Moreover, the acetyl group at C-34 of structure **1** formed a hydrogen bond with the imidazole nitrogen of His-419.

The crystal structure for the mouse was prepared by SYBYL-X 2.0. The docking score of structure **1** (8.6) was significantly higher than the ginsenoside compound K (4.2). The ginsenoside compound K ligand occupies a proportionally large volume of the cavity space within the ligand-binding pocket of LXRα (Figure 6A). The experimental result showed ginsenoside compound K and structure **1** is primarily bound to LXRα through hydrogen bonding via the hydroxyl group present on the molecule. A hydrogen bond was predicted between the hydroxyl group at C-36 of compound K and the carboxyl group of His-417, and the hydroxyl group at C-12 of compound K formed a hydrogen bond with the hydroxyl group ofThr-300 (Figure 6B). Structure **1** displays different bonding modes, the ester group at C-3 of structure **1** formed a hydrogen bond with the amide group of Asn-223, and the carboxyl group at C-3 of structure **1** formed a hydrogen bond with the amide group of Leu314.Moreover, the acetyl group at C-34 of **1** formed a hydrogen bond with the imidazole nitrogen of His-419 (Figure 6C).

### 2.7. Discussion

Oil Red O staining experiment results indicated that the ginsenoside compound K derivatives caused a significant decrease in lipid deposition in the macrophage-derived foam cells. Ginsenoside compound K and the derivatives showed an elevation of luciferase activity for LXRα at different levels and did not show significant activation of LXRβ. Ginsenoside compound K and structure **1** did not adopt a uniform binding way, as did other steroid agonists [16,17]. Hence, the ligand docking study has provided insight into the binding affinity to LXRα receptors. The characterization of LXRα as a regulator of reverse cholesterol transport is well known. Studies show that the activation of LXRα can up-regulate multiple downstream genes, including ABCA1 and ABCG1 in macrophages as efflux transporters [18], and ABCG5 and ABCG8 in enterocytes as excretion transporters [19]. In the present research into macrophages, we used ABCA1 mRNA levels as the marker of the activation degree of LXRα. The results of cell models showed structure **1** presented the best bioactivity, highest ABCA1 mRNA level, and lowest cellular cholesterol ester level among the compound K derivatives. Our results presented here suggest that all of the structures unambiguously enhanced the activation of LXRα, but the activation potency gradually declines along with the growth of the fatty acid carbon chain, and, quite the opposite, that the cytotoxicity of structures grows gradually following the growth of the fatty acid carbon chain. Among all tested structures, structure **1** exhibited the best potency and lower cell toxicity.

## 3. Design and Syntheses

**Scheme 1:**
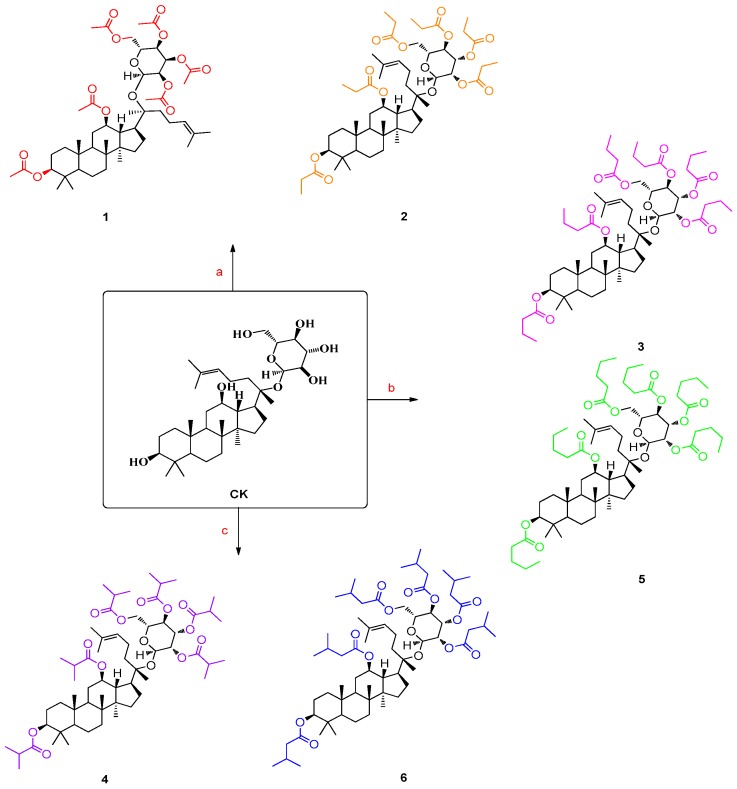
Synthesis of ginsenoside compound K derivatives structures **1**–**6**. Reagents and conditions (**a**) short chain fatty anhydride–pyridine, 60 °C,24 h; (**b**) short-chain, fatty anhydride, pyridine and DMAP (4-dimethylaminopyridine), 80 °C, 24 h; (**c**) short-chain fatty anhydride, pyridine and DMAP, 90 °C, 24 h.

Our previous results have showed that compound K could attenuate the formation of atherosclerosis in mice via activating LXRα. On the basis of previous literature, it can be seen that short-chain, fatty-acid-modified molecules markedly increase water solubility [20,21] and enhance the cellular uptake of molecules [22,23]. An attractive feature of these structures’ decomposition of secondary metabolites is the low toxicity natural by products [24]. Therefore, we designed various short-chain, fatty-acid-modified ginsenoside compound K analogues (Scheme 1). Preliminary results showed that structure **1** exhibited better efficacy for the activation of ABCA1 mRNA than ginsenoside compound K. Thus, we synthesized a series of short-chain, fatty-acid-modified ginsenoside compound K derivatives (**2**–**6**) for further exploration of the structure-activity relationships.

## 4. Experimental

### 4.1. General

All the starting materials were of reagent grade. The solvents used for the isolation and purification of the structures were obtained from J&K Scientific LTD (Beijing, China). All reactions were carried out in oven-dried glassware under an argon atmosphere unless otherwise noted. All yields reported refer to the yields of the isolated structures. RAW264.7, HUVEC and HEK293 cells were obtained from the Type Culture Collection of the Chinese Academy of Sciences (Shanghai, China). CCK-8 reagents were obtained from Dojindo (Kumamoto, Japan), Oil Red O were obtained from Sigma-aldrich (St. Louis, USA), Total RNA Kit was obtained from Tiangen (Beijing, China), PrimeScript™ RT reagent Kit and SYBR^®^ Premix Ex Taq™ II were obtained from TaKaRa (Tokyo, Japan), REALPLEX were obtained from Eppendorf (Hamburg, Germany).^1^H-NMR and ^13^C-NMR spectra were recorded using a Varian Inova-600 spectrometer (600 MHz). High-resolution mass spectra were obtained with a MALDI-TOF (MALDI-7090, SHIMADZU) mass spectrometer. Silica gel TLC plates (Qing Dao Marine Chemical Factory, Qingdao, China) were used to monitor the progression of the reactions. Flash column chromatography was performed using silica gel (200–400 mesh size, Qing Dao Marine Chemical Factory, Qingdao, China).

### 4.2. General Synthetic Procedure for Structures ***1***–***6*** [25,26,27,28]

#### 4.2.1. Ginsenoside Compound K Derivative **1** (Structure **1**)

A solution of ginsenoside compound K (0.1 g, 1.6 μmol) in pyridine (1 mL) was mixed with Ac2O (1 mL), and the mixture was stirred at 60 °C for 24 h. Then the mixture was added to ddH_2_O (500 mL) and the product was isolated by extraction with dichloromethane (300 mL). The organic phase was washed with lye, brine, dried over anhydrous sodium sulfate, filtered, then Silica gel was added and concentrated under vacuum. After the evaporation of excess reagent, the residue was subjected to column chromatography on silica gel using PE/EtOAc (5/1, *v*/*v*) to yield white powder (0.12 g, 13.7 μmol, 85%).

^1^H-NMR (600 MHz, CDCl_3_) δ 5.17 (t, *J* = 9.5 Hz, 1H), 5.02–4.96 (m, 2H), 4.93–4.88 (m, 1H), 4.81 (td, *J* = 10.9, 5.1 Hz, 1H), 4.65 (d, *J* = 7.9 Hz, 1H), 4.46 (dd, *J* = 11.0, 4.5 Hz, 1H), 4.14–4.06 (m, 2H), 3.66–3.61 (m, 1H), 2.09–1.99 (m, 7H), 1.97 (d, *J* = 8.9 Hz, 3H) (Supplementary Figure S1).

^13^C-NMR (151 MHz, CDCl_3_) δ 175.36, 174.99, 174.80, 174.23, 173.86, 173.39, 134.73, 128.49, 98.22, 86.86, 84.77, 79.02, 77.19, 75.98, 75.03, 72.66, 66.13, 59.68, 56.60, 53.73, 52.36, 52.07, 49.27, 43.33, 42.31, 41.43, 40.66, 38.07, 35.11, 32.61, 30.93, 29.95, 28.40, 27.11, 26.50, 25.11, 24.57, 23.64, 23.52, 23.24, 23.09, 23.07, 21.76, 21.04, 20.38, 19.42, 19.20, 18.66 (Supplementary Figure S7).

MALDI-TOF-MS *m/z* calcd. for C_48_H_74_O_14_[M + Na]^+^ 897.51, found 897.674.

#### 4.2.2. Ginsenoside Compound K Derivative **2** (Structure **2**)

A solution of ginsenoside compound K (0.1 g, 1.6 μmol) in pyridine (1 mL) was mixed with propionic anhydride (1 mL), and the mixture was stirred at 60 °C for 24 h. Then the mixture was added to ddH_2_O (500 mL) and the product was isolated by extraction with dichloromethane (300 mL). The organic phase was washed with lye, brine, dried over anhydrous sodium sulfate, filtered, and then Silica gel was added and concentrated under vacuum. After the evaporation of excess reagent, the residue was subjected to column chromatography on silica gel using PE/EtOAc (5/1, *v*/*v*) to yield white powder (0.13 g, 13.6 μmol, 84%).

^1^H-NMR (600 MHz, CDCl_3_) δ 5.20 (t, *J* = 9.5 Hz, 1H), 5.00 (dd, *J* = 18.6, 8.9 Hz, 2H), 4.94 (dd, *J* = 9.6, 8.0 Hz, 1H), 4.83 (td, *J* = 10.9, 5.0 Hz, 1H), 4.65 (d, *J* = 7.9 Hz, 1H), 4.48 (dd, *J* = 11.5, 4.4 Hz, 1H), 4.14–4.07 (m, 2H), 3.74–3.61 (m, 2H), 2.40–2.15 (m, 12H), 1.09 (dddd, *J* = 26.8, 19.2, 11.5, 6.5 Hz, 17H), 0.95 (s, 3H), 0.91 (s, 3H), 0.86 (s, 3H), 0.83 (d, *J* = 3.1 Hz, 6H) (Supplementary Figure S2).

^13^C-NMR (151 MHz, CDCl_3_) δ174.18, 174.16, 173.73, 173.65, 172.92, 172.42, 131.39, 124.25, 109.82, 94.54, 83.01, 80.15, 74.77, 72.96, 71.66, 71.51, 68.76, 68.50, 62.37, 55.74, 53.02, 49.65, 47.21, 45.29, 39.41, 39.07, 38.35, 37.87, 36.88, 34.32, 31.73, 29.03, 28.11, 27.99, 27.90, 27.41, 27.38, 27.35, 27.24, 26.30, 25.58, 23.47, 22.87, 22.51, 21.96, 18.07, 17.63, 16.36, 15.99, 15.31, 9.26, 9.07, 9.00, 8.98, 8.96, 8.89 (Supplementary Figure S8).

MALDI-TOF-MS *m/z* calcd. for C_54_H_86_O_14_[M + Na]^+^ 981.60, found 981.313.

#### 4.2.3. Ginsenoside Compound K Derivative **3** (Structure **3**)

A solution of ginsenoside compound K (0.1 g, 1.6 μmol) and DMAP (0.01 g, 0.08 mmol) in pyridine (1 mL) was mixed with butyric anhydride (1 mL), and the mixture was stirred at 80 °C for 24 h. Then the mixture was added to ddH_2_O (500 mL) and the product was isolated by extraction with dichloromethane (300 mL). The organic phase was washed with lye, brine, dried over anhydrous sodium sulfate, filtered, then Silica gel was added and concentrated under vacuum. After the evaporation of excess reagent, the residue was subjected to column chromatography on silica gel using PE/EtOAc (5/1, *v*/*v*) to yield white powder (0.09 g, 8.6 μmol, 55%).

^1^H-NMR (600 MHz, CDCl_3_) δ 5.20 (t, *J* = 9.5 Hz, 1H), 5.01 (dd, *J* = 19.8, 10.0 Hz, 1H), 4.94 (dd, *J* = 9.6, 8.0 Hz, 1H), 4.82 (td, *J* = 10.8, 4.9 Hz, 1H), 4.64 (d, *J* = 7.9 Hz, 1H), 4.48 (dd, *J* = 11.2, 4.6 Hz, 1H), 4.13 (dd, *J* = 12.0, 2.1 Hz, 1H), 4.05 (dd, *J* = 12.1, 6.1 Hz, 1H), 3.65–3.59 (m, 1H), 2.45–2.10 (m, 12H), 1.65–1.56 (m, 18H), 1.04–0.71 (m, 33H) (Supplementary Figure S3).

^13^C-NMR (151 MHz, CDCl_3_) δ 173.40, 173.32, 172.87, 172.80, 172.06, 171.58, 131.46, 124.41, 94.69, 83.17, 80.27, 74.92), 72.93, 71.68, 71.53, 68.51, 62.36, 55.84, 53.11, 49.94, 47.28, 45.50, 39.49, 38.99, 38.43, 38.42, 37.89, 36.94, 36.81, 36.72, 35.95, 35.86, 35.80, 34.40, 31.84, 29.08, 27.95, 26.41, 25.68, 23.58, 22.94 , 22.10, 18.59, 18.26, 18.21, 18.20, 18.13, 18.12, 18.10, 17.72, 16.50, 16.13, 15.40, 13.73, 13.72, 13.63, 13.59, 13.58, 13.58 (Supplementary Figure S9).

MALDI-TOF-MS *m/z* calcd. for C_60_H_98_O_14_[M + Na]^+^ 1065.70, found1065.425.

#### 4.2.4. Ginsenoside Compound K Derivative **4** (Structure **4**)

A solution of ginsenoside compound K (0.1 g, 1.6 μmol) and DMAP (0.01 g, 0.08 mmol) in pyridine (1 mL) was mixed with isobutyric anhydride (1 mL), and the mixture was stirred at 90 °C for 24 h. Then the mixture was added to ddH_2_O (500 mL) and the product was isolated by extraction with 300 mL dichloromethane (300 mL). The organic phase was washed with lye, brine, dried over anhydrous sodium sulfate, filtered, then Silica gel was added and concentrated under vacuum. After the evaporation of excess reagent, the residue was subjected to column chromatography on silica gel using PE/EtOAc (5/1, *v*/*v*) to yield white powder (0.06 g, 5.6 μmol, 35%).

^1^H-NMR (600 MHz, CDCl_3_) δ 5.28 (t, *J* = 9.5 Hz, 2H), 5.05 (dd, *J* = 19.8, 10.0 Hz, 1H), 5.03 (dd, *J* = 9.6, 8.0 Hz, 1H), 4.97 (td, *J* = 10.8, 4.9 Hz, 1H), 4.46 (d, *J* = 7.9 Hz, 1H), 4.01 (dd, *J* = 12.0, 2.1 Hz, 1H), 3.67 (dd, *J* = 12.1, 6.1 Hz, 1H), 3.51 (m, 1H), 2.61–2.35 (m, 6H), 0.87–0.79 (m, 36H) (Supplementary Figure S4).

^13^C-NMR (151 MHz, CDCl_3_) δ173.55, 173.48, 173.03, 172.98, 172.20, 171.71, 131.11, 124.09, 94.51, 83.13, 80.17, 74.72, 72.71, 71.65, 71.52, 68.45, 62.29, 55.74, 52.97, 49.90, 47.11, 45.44, 39.46, 38.98, 38.36, 37.85, 36.91, 34.67, 34.53, 34.39, 33.78, 33.77, 33.71, 33.67, 31.80, 29.07, 27.95, 27.19, 26.87, 26.85, 26.77, 26.75, 25.68, 23.56, 22.94, 22.32, 22.30, 22.29, 22.20, 22.19, 22.16, 18.12, 17.74, 16.50, 16.14, 15.40, 13.79, 13.74, 13.72, 13.60 (Supplementary Figure S10).

MALDI-TOF-MS *m/z* calcd. for C_60_H_98_O_14_[M + H]^+^ 1066.70, found 1066.522.

#### 4.2.5. Ginsenoside Compound K Derivative **5** (Structure **5**)

A solution of ginsenoside compound K (0.1g, 1.6μmol) and DMAP (0.01g, 0.08mmol) in pyridine (1 mL) was mixed with valeric anhydride (1 mL), and the mixture was stirred at 80 °C for 24 h. Then the mixture was added to ddH_2_O (500 mL) and the product was isolated by extraction with dichloromethane (300 mL). The organic phase was washed with lye, brine, dried over anhydrous sodium sulfate, filtered, then Silica gel was added and concentrated under vacuum. After the evaporation of excess reagent, the residue was subjected to column chromatography on silica gel using PE/EtOAc (5/1, *v*/*v*) to yield white powder (0.08 g, 6.9μmol, 43%).

^1^H-NMR (600 MHz, CDCl_3_) δ 5.20 (t, *J* = 9.5 Hz, 1H), 5.01 (t, *J* = 9.7 Hz, 1H), 4.98 (s, 1H), 4.96–4.91 (m, 1H), 4.81 (td, *J* = 10.7, 4.8 Hz, 1H), 4.64 (d, *J* = 7.9 Hz, 1H), 4.47 (dd, *J* = 11.1, 4.6 Hz, 1H), 4.12 (dd, *J* = 12.0, 2.0 Hz, 1H), 4.05 (dd, *J* = 12.1, 6.0 Hz, 1H), 3.65–3.59 (m, 1H), 2.38–2.15 (m, 12H), 1.36–1.25 (m, 12H), 0.91–0.84 (m, 18H) (Supplementary Figure S5).

^13^C-NMR (151 MHz, CDCl_3_) δ 173.54, 173.46, 173.02, 172.98, 172.19, 171.72, 131.54, 124.42, 94.70, 83.17, 80.27, 74.93, 73.14, 71.67, 71.57, 68.56, 62.39, 55.86, 53.11, 49.96, 47.24, 45.53, 39.51, 39.01, 38.45, 37.90, 36.95, 34.71, 34.52, 34.43, 33.78, 33.76, 33.70, 33.66, 31.85, 29.10, 27.95, 27.23, 27.18, 26.87, 26.84, 26.76, 26.75, 26.72, 26.40, 25.65, 23.58, 22.96, 22.31, 22.29, 22.27, 22.19, 22.17, 22.15, 22.07, 18.12, 17.72, 16.49, 16.13, 15.43, 13.76, 13.76, 13.71, 13.69, 13.59, 13.58 (Supplementary Figure S11).

MALDI-TOF-MS *m/z* calcd. for C_66_H_110_O_14_ [M + Na]^+^ 1149.79, found 1149.492.

#### 4.2.6. Ginsenoside Compound K Derivative **6** (Structure **6**)

A solution of ginsenoside compound K (0.1 g, 1.6 μmol) and DMAP (0.01 g, 0.08 mmol) in pyridine (1 mL) was mixed with butyric anhydride (1 mL), and the mixture was stirred at 90 °C for 24h. Then the mixture was added to ddH_2_O (500 mL) and the product was isolated by extraction with dichloromethane (300 mL). The organic phase was washed with lye, brine, dried over anhydrous sodium sulfate, filtered, then Silica gel was added and concentrated under vacuum. After the evaporation of excess reagent, the residue was subjected to column chromatography on silica gel using PE/EtOAc (5/1, *v*/*v*) to yield white powder (0.09 g, 8.0 μmol, 50%).

^1^H-NMR (600 MHz, CDCl_3_) δ 5.21 (t, *J* = 9.5 Hz, 1H), 5.01 (dd, *J* = 19.2, 9.4 Hz, 2H), 4.93 (dd, *J* = 9.6, 7.9 Hz, 1H), 4.82 (td, *J* = 10.7, 4.8 Hz, 1H), 4.63 (d, *J* = 7.9 Hz, 1H), 4.48 (dd, *J* = 11.4, 4.7 Hz, 1H), 4.17 (dd, *J* = 12.1, 2.0 Hz, 1H), 4.00 (dd, *J* = 12.1, 6.0 Hz, 1H), 3.64–3.58 (m, 1H), 2.23–1.95 (m, 15H), 1.02–0.79 (m, 40H) (Supplementary Figure S6).

^13^C-NMR (151 MHz, CDCl_3_) δ 172.85, 172.73, 172.32, 172.11, 171.46, 170.98, 131.42, 124.44, 94.71, 83.24, 80.29, 74.90, 72.75, 71.69, 71.48, 68.55, 62.31, 55.85, 53.11, 49.99, 47.29, 45.53, 43.99, 43.98, 42.98, 42.95, 39.51, 38.71, 38.42, 37.83, 36.93, 34.41, 31.90, 29.68, 29.08, 27.96, 26.48, 25.75, 25.73, 25.54, 25.51, 25.30, 25.15, 25.04, 23.60, 22.96, 22.57, 22.48, 22.44, 22.42, 22.41, 22.40, 22.40, 22.39, 22.38, 22.37, 22.36, 22.33, 22.31, 22.13, 18.18, 18.13, 17.76, 16.52, 16.12, 15.42 (Supplementary Figure S12).

MALDI-TOF-MS *m/z* calcd. for C_66_H_110_O_14_ [M + Na]^+^ 1149.79, found 1149.439.

### 4.3. Water Solubility Measurements

1 mg Protopanoxadiol (PPD) was placed into a vial containing 1mL of methanol, and 2 mg of ginsenoside compound K or ginsenoside compound K derivative structure **1** was placed separately into a vial containing 1 mL ddH_2_O. The vials were sealed and shaken for 6 h at 25 °C until reaching equilibrium. After centrifugation at 12,000× *g* for 10 min, the supernatant was filtered through a 0.22 μm filter. The measured PPD solution (0.1 mL) was blended to 0.9 mL ginsenoside compound K or structure **1** filtrate well, respectively. The concentrations of ginsenoside compound K and structure **1** in the filtrate were determined by HPLC (Ac:H_2_O = 60:40; λ = 203 nm). Experiments were performed in triplicate.

### 4.4. Formation of Foam Cells (Oil Red O Staining) [29]

RAW264.7 cells were seeded in 24-well plates covered by glass slides at 1 × 10^5^/mL, and incubated with 100 μg/mL ox-LDL and different doses of tested structures for another 24 h. The cells were treated with ox-LDL (ox-low density lipoprotein) and DMSO solution (1:1000) as the model group. The cells were gently washed with PBS (phosphate buffer saline) three times and fixed with 4% paraformaldehyde for 30 min, and then subsequently stained with Oil Red O for 1 h. The accumulated lipid droplets in the macrophages were visualized using a Nikon Eclipse 90i light microscope (Nikon Instruments, New York, NY, USA). Oil Red O stained cellular cholesteryl ester was extracted from the foam cells by isopropanol, and quantified by the optical density values at 500 nm. The optical density values were calculated relative to the model group. The experiments were performed in octuplicate.

### 4.5. ABCA1 mRNA Expression in RAW264.7 [4]

RAW264.7 cells were seeded in 12-well plates at 1 × 106mL, then treated with a final concentration 10μM of the tested structures for 24 h after cell adherence. Total RNAs were extracted with RNA simple Total RNA Kit, and the cDNA was synthesized with the Prime Script™ RT reagent Kit with gDNA Eraser. Real-time PCR was performed using SYBR^®^ Premix Ex Taq™ II on a REALPLEX real-time PCR reaction system under the following conditions: 30 s at 95 °C, 40 cycles at 95 °C for 5 s and 60 °C for 30 s. Primers for mouse β-actin were 5′-ATTGAACATGGCATTGTTACC-3′ and 5′-GGCATACAGGGACAGCACAGC-3′; for mouse ABCA1 were 5′-ACATCCTCGTCCATTAAGCC-3′ and 5′-AACTCTGGCACACTCATTGC-3′. The fold increase relative to control samples was determined by the 2-ΔΔCt (cycle threshold) method, and the Ct values were normalized to the expression levels of β-actin. Experiments were performed in quintuplicate.

### 4.6. Cellular Toxicity

Cellular toxicity assays were carried out by the CCK-8 method [30] on RAW264.7 and HUVEC cells. Cells were seeded in 96-well plates at 3 × 10^4^/mL per well in RPMI-1640 containing 10% fetal bovine serum, then treated with different concentrations(10, 30 and 100 μM) of the tested structures for another 24 h after cell adherence. Meanwhile, one group of cells was given DMSO (1‰) as vehicle control. CCK-8 reagents (10 μL/well) were added into the wells. Cells were incubated 37 °C for 1 h, and the optical density values were measured at 450 nm by the microplate reader. The survival rates of the treated cells were calculated relative to the control group. Experiments were performed in triplicate.

### 4.7. Luciferase Reporter Assay

HEK293 cells, with 1 × 10^5^ cells/well in 96-well plates, were transfected with hLXREx3TK-Luc as a reporter, and pCMX-hLXRα or pCMX-hLXRβ as an expression vector, respectively. pSV-β-galactosidase was used to normalize the transfection efficiencies. The plasmids were transfected into the cells with Lipofectamine 3000 Reagent (Thermo Fisher Scientific, Waltham, MA, USA). After 24 h incubation, cells were treated with compound K derivatives (10 μM), or vehicle (1‰ DMSO) for 24 h. GW3965 (10 μM) was used as a positive control. Then the cells were lysed, and the luciferase and β-galactosidase activities were detected by Varioskan LUX (Thermo Fisher Scientific, Waltham, MA, USA). The results were presented as relative luciferase activity.

## 5. Conclusions

In conclusion, a new class of ginsenoside compound K derivatives was synthesized and evaluated regarding the activation of LXRα. All of the structures of this novel class of ginsenoside compound K derivatives were able to enhance the activation of LXRα. Especially structure **1** and structure **2** were identified as highly potent (cholesteryl ester contents: 41.51% and 37.74%) and as having low cytotoxicity. Further experiments proved that structures **1**, **2** and **4** obviously promoted ABCA1 mRNA expression (expression of ABCA1 mRNA: 319%, 278% and 259%) via LXRαactivity evaluation, and sequentially increased the level of reverse cholesterol transport. Among all the investigated structures, structure **1** exhibited the best potency (cholesteryl ester contents: 41.51%; expression of ABCA1 mRNA: 319%) and low cytotoxicity, this result provides a foundation for further modification of compound K research.

## Supplementary Materials

The supplementary materials are available online.
