# Supplementary material for: Synthesis and Biological Evaluation of Ginsenoside Compound K Derivatives as a Novel Class of LXRα Activator
Source: Molecules. 2017 Jul 24;22(7):1232. doi: 10.3390/molecules22071232 (PMC6152260; doi:10.3390/molecules22071232)
Supplement: Supplementary file 1 [file molecules-22-01232-s001.pdf]

^{1}H NMR of ginsenoside compound K derivatives

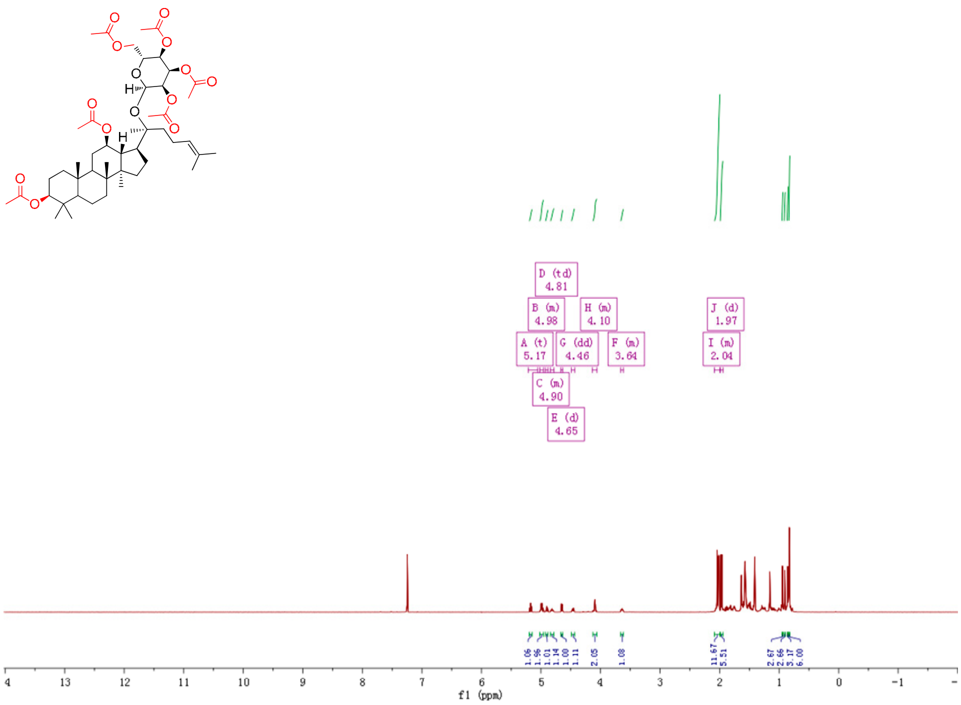

Figure 1. ^{1}H NMR of structure 1

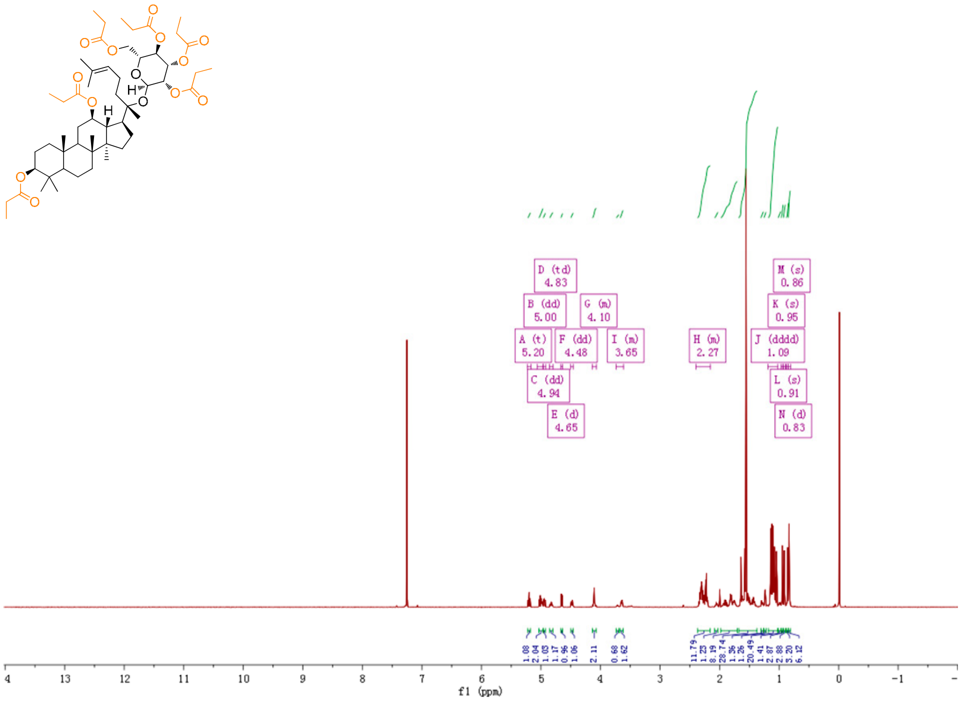

Figure 2. ^{1}H NMR of structure 2

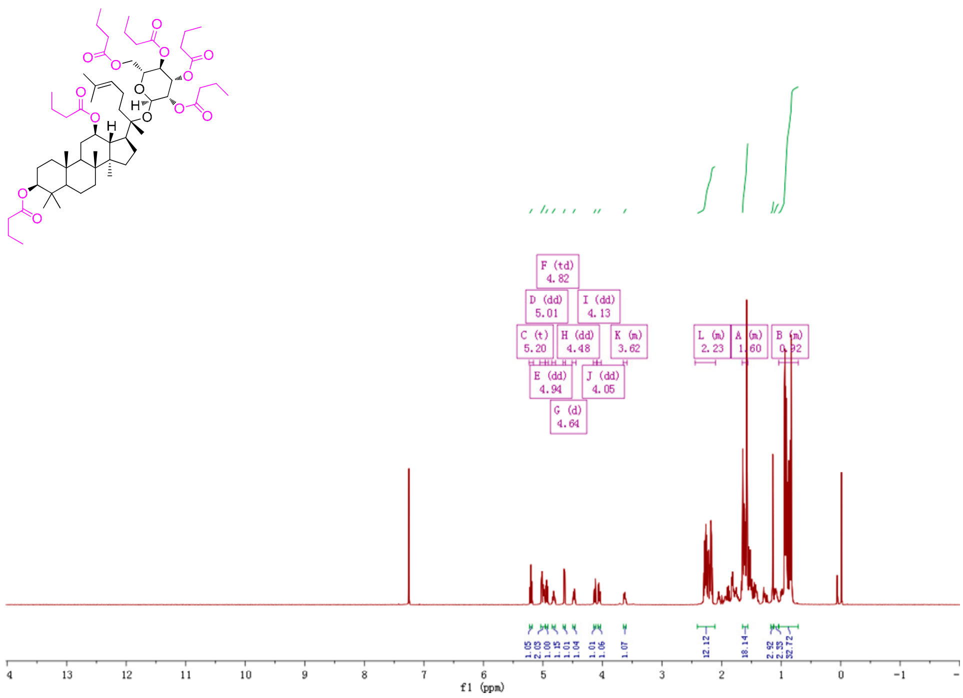

Figure 3. ^{1}H NMR of structure 3

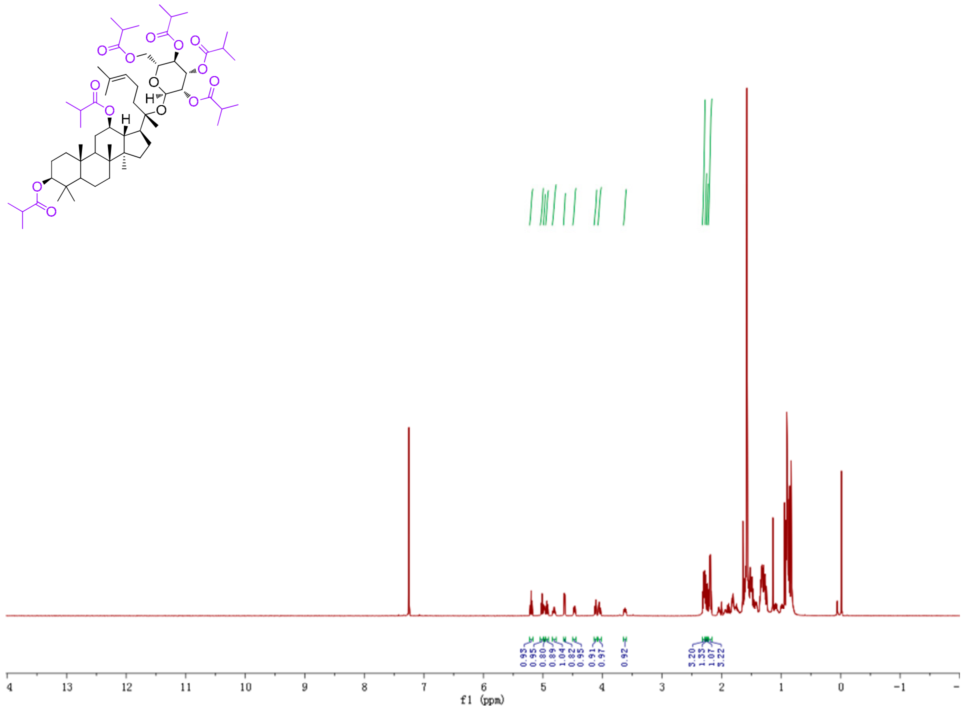

Figure 4. ^{1}H NMR of structure 4

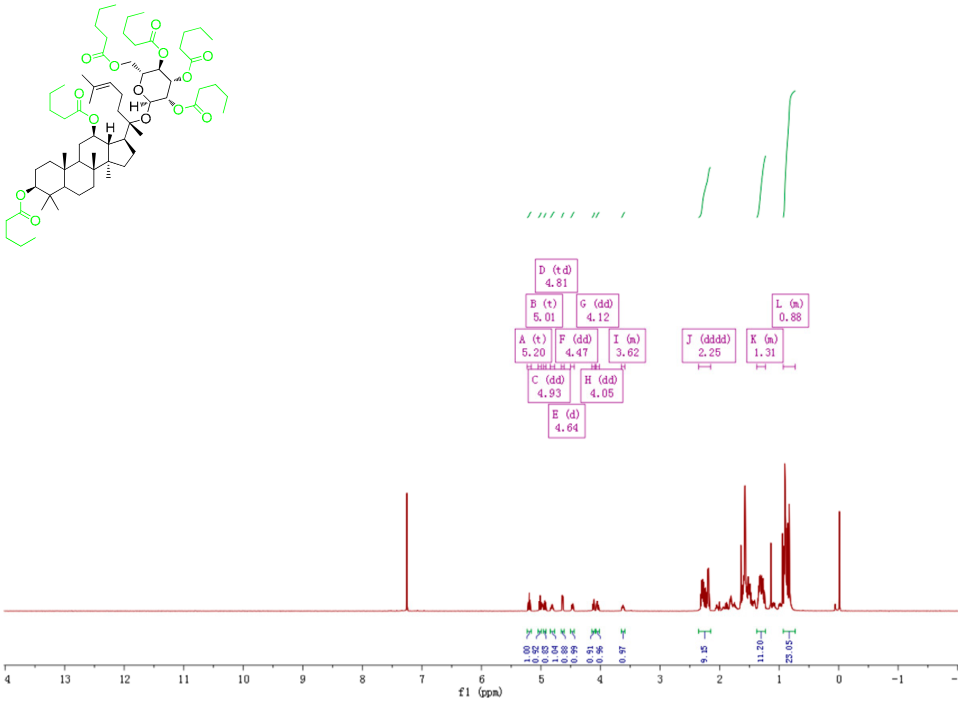

Figure 5. ^{1}H NMR of structure 5

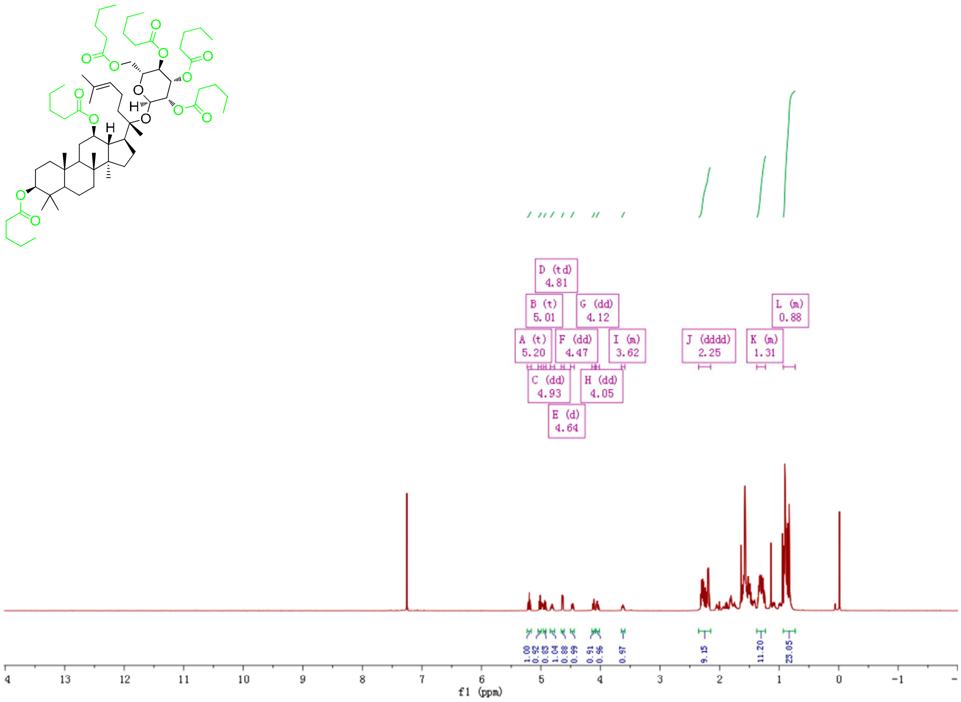

Figure 6. ^{1}H NMR of structure 6

^{13}C NMR of ginsenoside compound K derivatives

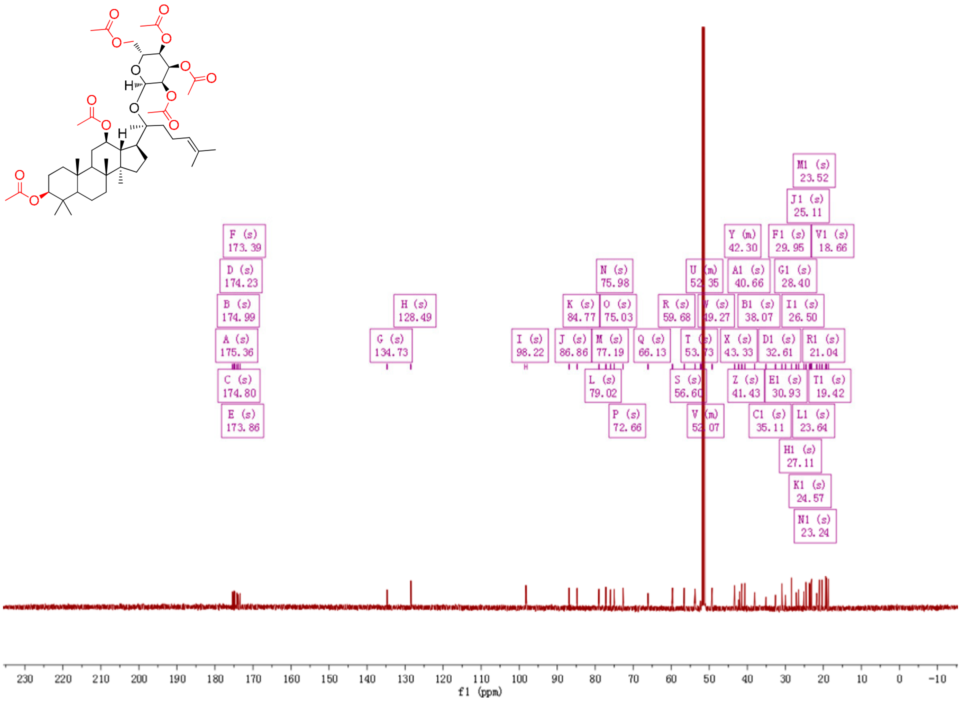

Figure 7. ^{13}C NMR of structure 1

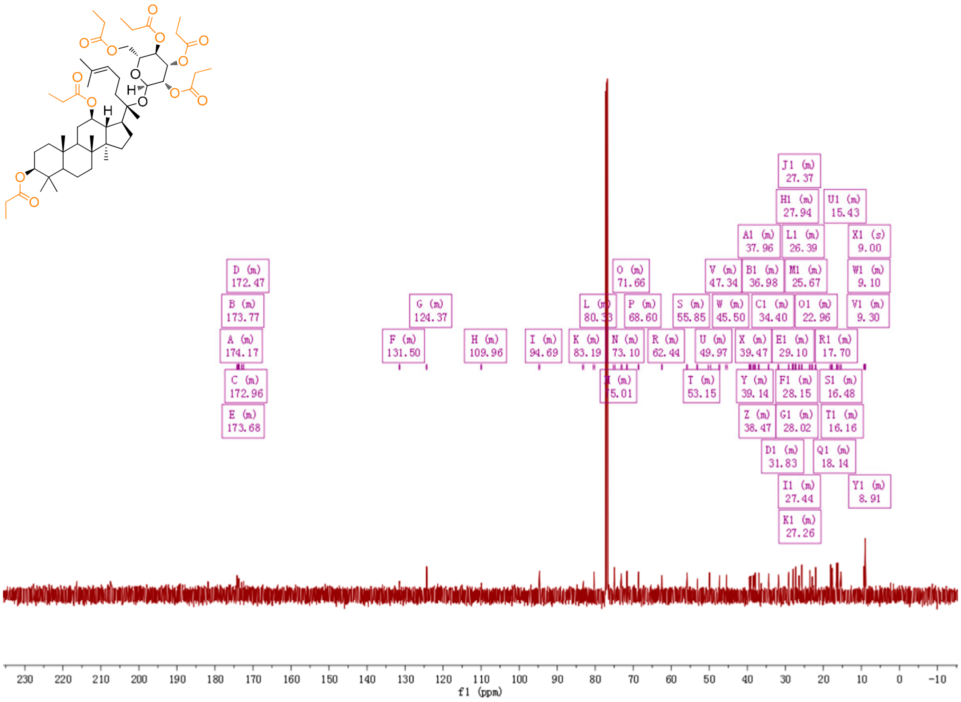

Figure 8. ^{13}C NMR of structure 2

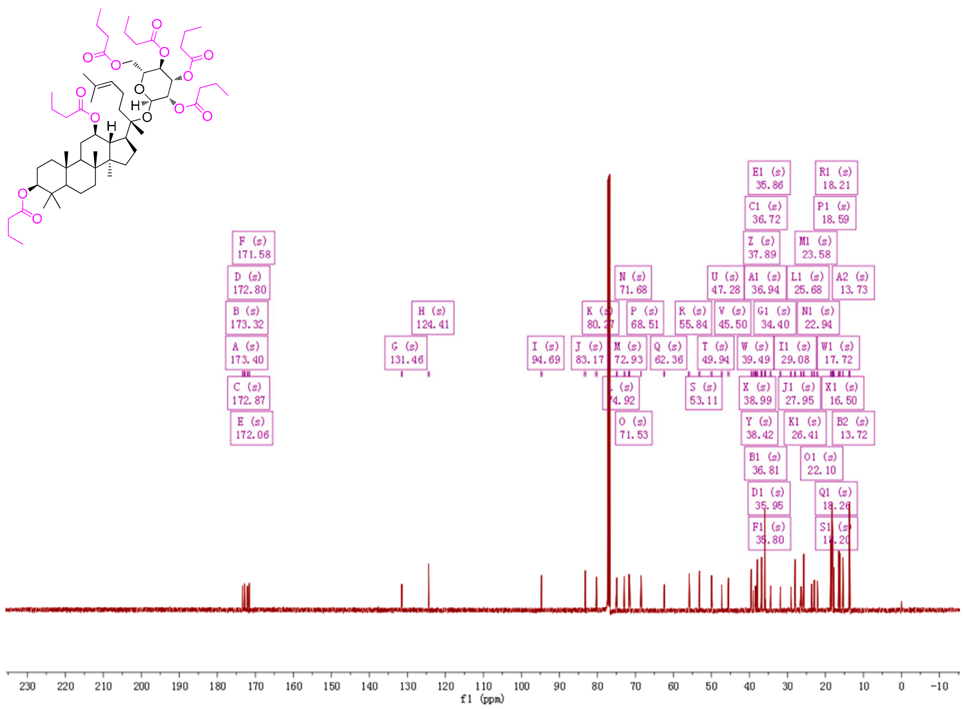

Figure 9. ^{13}C NMR of structure 3

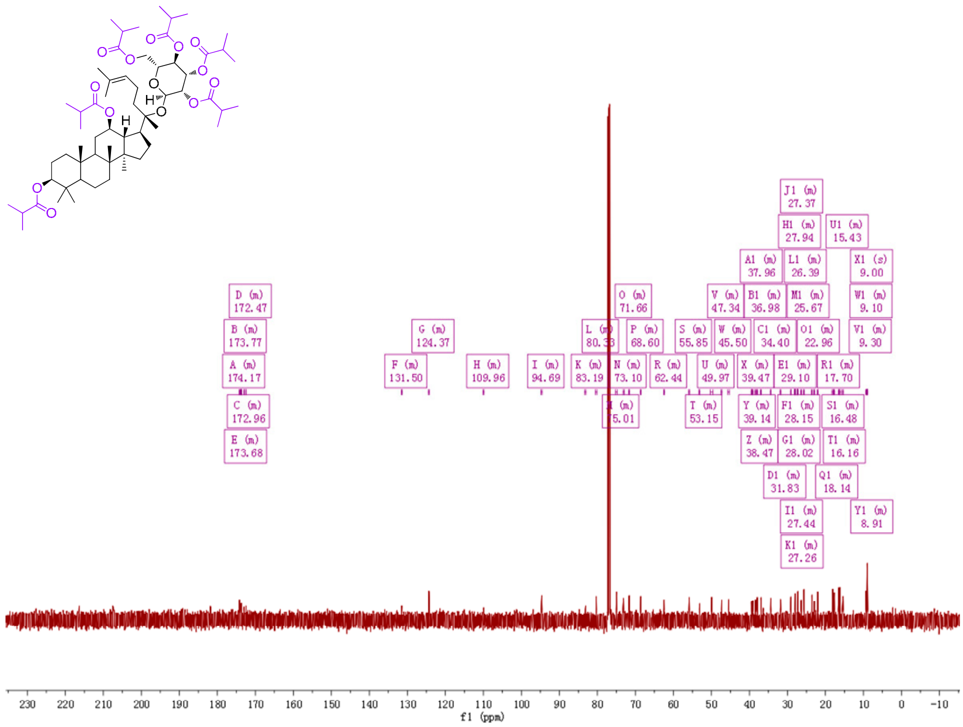

Figure 10. ^{13}C NMR of structure 4

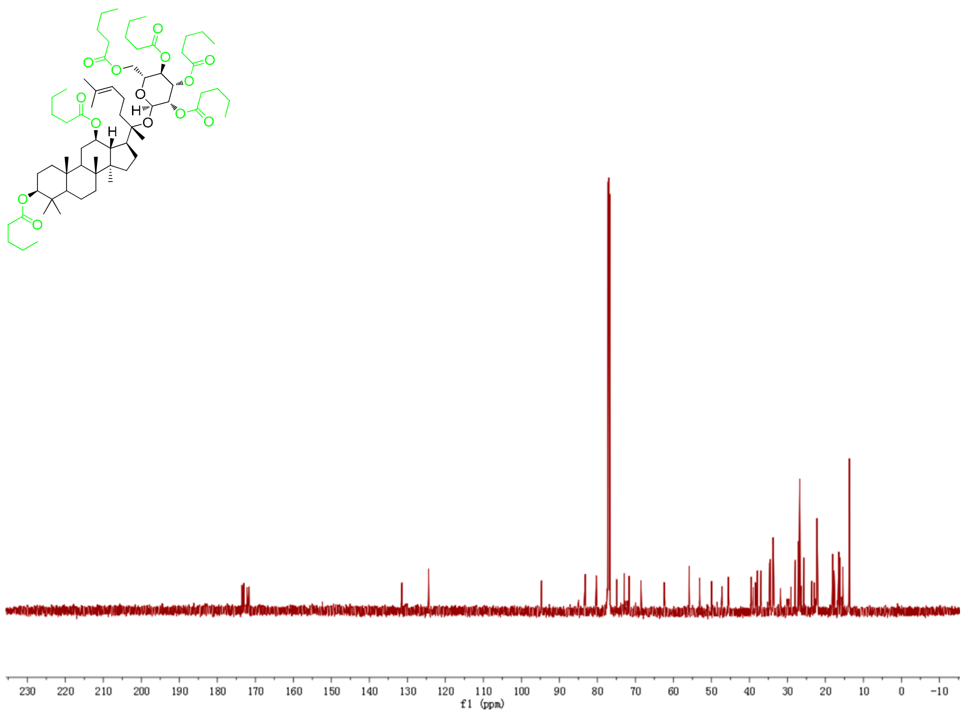

Figure 11. ^{13}C NMR of structure 5

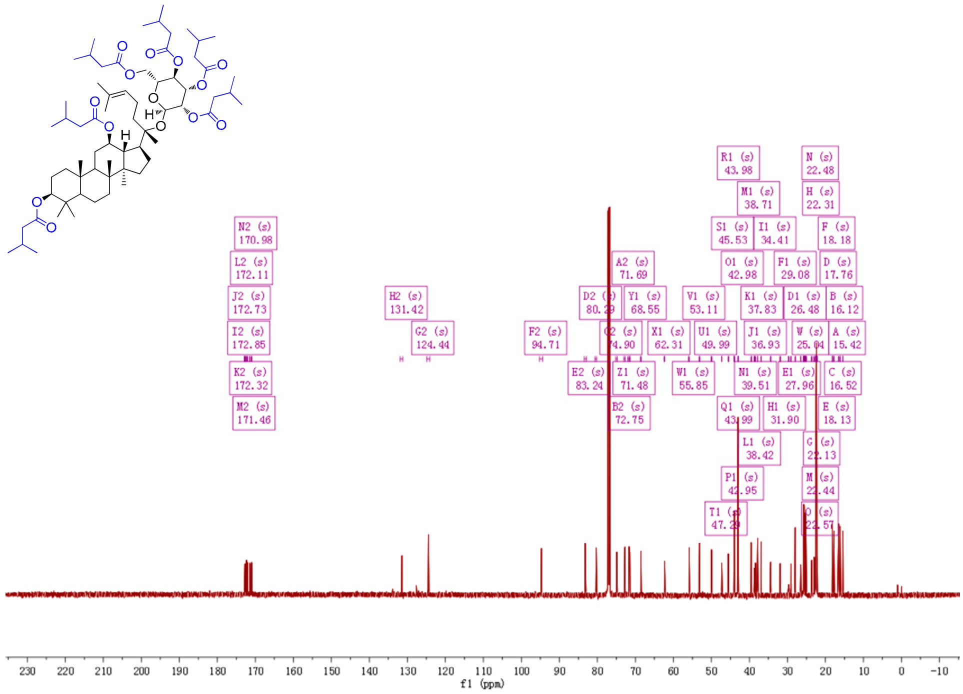

Figure 12. ^{13}C NMR of structure 6
